# Directing Differentiation of Pluripotent Stem Cells Toward Retinal Pigment Epithelium Lineage

**DOI:** 10.5966/sctm.2016-0088

**Published:** 2016-08-24

**Authors:** Parul Choudhary, Heather Booth, Alex Gutteridge, Beata Surmacz, Irene Louca, Juliette Steer, Julie Kerby, Paul John Whiting

**Affiliations:** ^1^ Pfizer Neuroscience and Pain Research Unit, Great Abington, Cambridge, United Kingdom

**Keywords:** Retinal pigment epithelium, Stem cells, Directed differentiation, Activin, Bone morphogenetic protein

## Abstract

Development of efficient and reproducible conditions for directed differentiation of pluripotent stem cells into specific cell types is important not only to understand early human development but also to enable more practical applications, such as in vitro disease modeling, drug discovery, and cell therapies. The differentiation of stem cells to retinal pigment epithelium (RPE) in particular holds promise as a source of cells for therapeutic replacement in age‐related macular degeneration. Here we show development of an efficient method for deriving homogeneous RPE populations in a period of 45 days using an adherent, monolayer system and defined xeno‐free media and matrices. The method utilizes sequential inhibition and activation of the Activin and bone morphogenetic protein signaling pathways and can be applied to both human embryonic stem cells and induced pluripotent stem cells as the starting population. In addition, we use whole genome transcript analysis to characterize cells at different stages of differentiation that provides further understanding of the developmental dynamics and fate specification of RPE. We show that with the described method, RPE develop through stages consistent with their formation during embryonic development. This characterization— together with the absence of steps involving embryoid bodies, three‐dimensional culture, or manual dissections, which are common features of other protocols—makes this process very attractive for use in research as well as for clinical applications. Stem Cells Translational Medicine *2017;6:490–501*

**Significance Statement**

This report describes a novel method of directed differentiation to generate retinal pigment epithelium (RPE) cells from pluripotent stem cells. The employed method is based on adherent monolayer culture using xeno‐free conditions and manipulation of the Activin and bone morphogenetic protein signaling pathway using small molecules and recombinant proteins. Whole genome microarray analysis was performed to characterize the differentiation process and understand the developmental path of RPE generation in vitro. This method can be applied for generation of RPE for research as well as for clinical applications.

## Introduction

Age‐related macular degeneration (AMD) is a leading cause of irreversible vision loss among the elderly population in the developed world 1. The primary cell type affected in this disease is the retinal pigment epithelium (RPE). RPE cells are situated as a monolayer beneath the photoreceptors and perform several important functions to maintain the visual cycle, for example, metabolism and storage of retinoid, phagocytosis of rod outer segments, absorption of scattered light, barrier activity, and ion transport 2. Degeneration of RPE in AMD results in subsequent loss of photoreceptors, leading to loss of vision. AMD manifests in two forms: the exudative or wet type and the dry type 3. Wet AMD is characterized by neovascularization of the choroid and can be controlled with intravitreal injections of anti‐vascular endothelial growth factor (VEGF) antibodies, small molecule inhibitors, or both. The dry type is more common, representing the majority of individuals with AMD, for which there are no therapies available at present. An approach that is currently under substantial investigation is the replacement of dysfunctional, diseased RPE with cells derived in vitro from stem cell differentiation. Preclinical as well as early clinical reports suggest that this strategy is safe and allows the newly transplanted RPE to effectively perform functions such as phagocytosis, fluid and nutrient transport, and photoreceptor support, leading to potential restoration of vision 4, 5, 6, 7. In addition, there are many features that make the eye an attractive organ for cell replacement and regenerative therapy approaches, such as the requirement of fewer cells for therapeutic intervention in comparison with other larger organs, accessibility and ease of monitoring, potential immune privilege, and presence of the retinal‐blood barrier, leading to separation from systemic circulation 8. Therefore, there has been considerable interest in developing protocols to generate RPE cells from pluripotent stem cells with high efficiency and purity to use for transplantation as well as to study AMD disease mechanisms.

Although several protocols describing differentiation of RPE from human embryonic stem cells (hESC) and human induced pluripotent stem cells (hiPSC) have been described, they often have limitations, including low yield, high variability, or steps involving embryoid bodies, cysts, or eye cup structures, together with manual excision of pigmented cells 9, 10. These limitations are undesirable for scale‐up and transfer to a good manufacturing practice (GMP) clinical manufacturing setting. Therefore, we aimed to develop a robust, adherent monolayer‐based directed differentiation protocol to generate RPE from pluripotent stem cells with high efficiency, with high yield, and in short time scales. We have applied knowledge of development of the vertebrate nervous system, in which temporal and spatial gradients of signaling molecules interact to regulate tissue formation, to devise an in vitro protocol for RPE differentiation that mimics developmental cues. The bone morphogenetic protein (BMP) and Activin/Nodal signaling pathways play important roles in development and specification of RPE fate. First, the neuroectoderm is specified by inhibition of BMP and Activin/Nodal signaling 11, 12, which then further differentiates into eye field and RPE in a process requiring sequential activation of BMP and Activin signaling 9, 13, 14, 15, 16. We have utilized this feature of dynamic activation and repression of these pathways for development of our method. Furthermore, we perform microarray profiling to understand the identity of the cells at the population level across all stages of our protocol. This allows us to identify key features of the differentiation as well as understand purity of the population, which can be used to design strategies for quality control and process development for clinical manufacturing applications.

## Materials and Methods

### Directed Differentiation of Pluripotent Stem Cells Toward RPE

**Figure 1:**
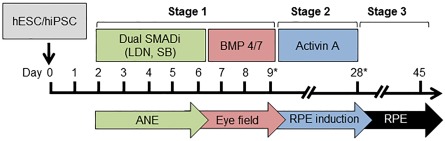
Directed differentiation of stem cells to retinal pigment epithelium. Schematic of the directed differentiation protocol described in this study. ∗, replating steps. Abbreviations: ANE, anterior neuroectoderm; BMP, bone morphogenetic protein; RPE, retinal pigment epithelium; hESC, human embryonic stem cells; hiPSC, human induced pluripotent stem cells; RPE, retinal pigment epithelium; SMADi, inhibitor of SMAD pathway.

All work was carried out in a sterile biological safety cabinet. hESC (SHEF1; WA26) or hiPSC (AD3.1; SendaiF, which refers to erythroblasts reprogrammed using the CytoTune‐iPS Reprogramming kit; Thermo Fisher Scientific Life Sciences, Waltham, MA, http://www.thermofisher.com) were routinely cultured on human recombinant vitronectin (Thermo Fisher) in Essential 8 medium (Thermo Fisher) or on hESC‐qualified Matrigel (Corning, Manassas, VA, https://www.corning.com) in mTeSR1 medium (STEMCELL Technologies, Vancouver, BC, Canada, https://www.stemcell.com). A schematic of the differentiation process is shown in Figure 1. Pluripotent cells were treated with 10 μM Y‐27632 ROCK inhibitor (Tocris Bioscience, Bristol, U.K., https://www.tocris.com) for 1 hour prior to dissociation. The cells were dissociated with Accutase (Thermo Fisher) and passed through a 40‐μm cell strainer to remove larger aggregates. Cells were seeded at a density of 240,000 cells per cm^2^ onto T25 flasks (Nunclon; Thermo Fisher) or 96‐well plates (Corning CellBIND) coated with matrices, for example, growth factor reduced Matrigel (Corning) or human plasma‐derived fibronectin (Sigma‐Aldrich, St. Louis, MO, https://www.sigmaaldrich.com) in E8 or TeSR2 (STEMCELL Technologies) medium containing 5 µM Y‐27632. The next day (day 1), culture medium was changed to E8 or TeSR2 without Y‐27632. On day 2, cells were switched to Induction Medium 1: Dulbecco's modified Eagle's medium (DMEM) KSR‐XF (KnockOut DMEM medium [Thermo Fisher], supplemented with 20% KnockOut Serum Replacement Xeno‐Free [Thermo Fisher], 1% β‐Mercaptoethanol [Sigma‐Aldrich], 1% GlutaMax [Thermo Fisher], and 1% non‐essential amino acid solution [Thermo Fisher]), supplemented with two inhibitors (LDN/SB): 1 μM LDN‐193189 (Stemgent, Cambridge, MA, https://www.stemgent.com) and 10 µM SB‐431542 (Sigma‐Aldrich). Induction Medium 1 was replenished daily for 4 days (day 2 to day 5). On day 6, culture medium was switched to Induction Medium 2: DMEM KSR‐XF, supplemented with 100 ng/ml BMP 4/7 (R&D Systems, Minneapolis, MN, https://www.rndsystems.com) for 3 days. The culture medium was replenished every day up to day 9. The period from day 0 —that is, at the point of seeding pluripotent cells to day 9—is referred to as stage 1 for the remainder of this article. On day 9, the cells were replated. The cultures were pretreated with 10 μM Y‐27632 for 35 min, dissociated by using Accutase, passed through a 40‐μm cell strainer, and seeded at a density of 320,000 cells per cm^2^ on Matrigel‐ or CELLStart‐ (Thermo Fisher) coated surfaces. Cells were treated with Induction Medium 3: DMEM KSR‐XF, supplemented with 100 ng/ml Activin A (ActA; R&D Systems). The Induction Medium 3 was replenished three times a week until day 28. This period of the protocol, from day 9 to day 28, is referred to as stage 2. On day 28, cells were dissociated with Accutase, passed through a 70‐µm cell sieve and plated at a density of 100,000 cells per cm^2^ on CELLStart‐coated surfaces. The replated cells were replenished with DMEM KSR‐XF twice a week for at least 14 days, that is, until day 42, before the identity and purity of the cells were assessed by various methods. This stage of the protocol, from day 28 to day 42 and beyond, is referred to as stage 3. For culture on polyester membrane in transwell culture inserts, cells from the end of stage 3 were dissociated and replated on Vitronectin‐coated polyester transwells at a density of 360,000 cells per cm^2^. The replated cells were replenished with DMEM KSR‐XF twice a week, and the media from the top and bottom compartments were kept separate. Spent medium was collected for further analysis of secreted factors by using commercially available enzyme‐linked immunosorbent assay kits (MS6000, human VEGF pigment epithelium‐derived factor [PEDF] duplex assay) on the Mesoscale discovery platform. Bead phagocytosis assay was performed with polystyrene microspheres (1.0‐μm Red FluoSpheres; Thermo Fisher), following a published protocol 17. RPE were generated from hESC by spontaneous differentiation with a protocol described previously 18.

### RNA Extraction, cDNA Synthesis, and Quantitative Polymerase Chain Reaction

Total RNA was extracted from RPE cells using the RNeasy Mini or Micro Kit (Qiagen, Hilden, Germany, https://www.qiagen.com) with on‐column DNase digestion. cDNA was synthesized using the High Capacity cDNA Synthesis kit (Thermo Fisher). Individual gene expression was assessed using predesigned Taqman assays (Thermo Fisher) and the reactions were carried out on the CFX96 iCycler platform (Bio‐Rad, Raleigh, NC, https://www.bio‐rad‐antibodies.com). Gene expression in all instances was quantified by the relative quantification method of 2^−ΔΔCt^ and normalized to geometric means of at least two housekeeping genes.

### Microarray Analysis

mRNA was hybridized on Illumina HT‐12v4 BeadChips according to the manufacturer's instructions. The microarray data are available in the ArrayExpress database under accession number E‐MTAB‐4259. Raw data were log^2^ transformed and quantile normalized with R/Biocondcutor and the lumi and beadarray packages. Principal components analysis (PCA) was performed using the pcaMethods package. Differential expression between time points was performed using the limma package with a multiple testing adjusted *p* value cutoff of *p* < .01.

### Immunocytochemistry

Samples were fixed in 4% paraformaldehyde in phosphate‐buffered saline (PBS) for 15 min followed by blocking and permeabilization using 0.3% Triton X‐100 in PBS and 10% normal donkey serum. Primary antibodies used in this study are anti‐PMEL17 (Dako, Carpinteria, CA, http://www.dako.com, M0634), anti‐ZO1 (Thermo Fisher, 187430), anti‐CRALBP (Affinity Biosciences, Cambridge, U.K., http://www.affbiotech.com, MA1‐813), anti‐OCT4 (Santa Cruz Biotechnology, http://www.scbt.com, sc‐8628), anti‐Pax6 (Covance, Princeton, NJ, http://www.covance.com, PRB‐278P), anti‐MITF (Thermo Fisher, MS‐772‐PABX), anti‐Otx2 (Merck Millipore, Darmstadt, Germany, http://www.emdmillipore.com, ab9566), anti‐Lhx2 (Santa Cruz Biotechnology, sc‐19344), and anti‐β Crystallin (Abcam, Cambridge, U.K., http://www.abcam.com, ab151722). Nuclei were counterstained with the nuclear dye Hoechst. Images were captured and analyzed on the ImageXpress platform (Molecular Devices, Sunnyvale, CA, https://www.moleculardevices.com) or an epifluorescent microscope (Carl Zeiss AG, Oberkochen, Germany, http://www.zeiss.com).

## Results

### Dual SMAD Inhibition Followed by BMP Pathway Activation Leads to Differentiation of Pluripotent Stem Cells to Eye Field

Previous work from our group has demonstrated the efficient induction of anterior neuroectoderm (ANE) from pluripotent stem cells using small molecule inhibitors of the bone morphogenetic protein (BMP) and Activin/Nodal signaling arms of the TGFβ pathway 11. Activin/Nodal signaling leads to phosphorylation of SMAD2/3 through the action of ALK4/5/7 kinases, whereas BMP signaling leads to phosphorylation of SMAD1/5/8 through ALK2/3 kinases. We have shown that use of 10 µM SB‐431642 (SB) and 1 µM LDN‐193189 (LDN) leads to effective inhibition of the Activin/Nodal and BMP signaling pathways, resulting in downstream “dual SMAD” inhibition. This directs the pluripotent stem cells toward the ANE fate efficiently and homogeneously. Furthermore, dual SMAD inhibition has been shown to increase RPE yield using the spontaneous differentiation method, indicating that this mechanism is involved in RPE differentiation 19. Therefore, we wanted to extend these observations to further direct the ANE state toward eye field specification and RPE formation in a controlled manner. Knowledge of developmental biology from zebrafish, chick, and rodent models informs us that the regionalization of the ANE toward the forebrain, and further specification of the forebrain cells toward eye field is achieved through the temporal induction of BMP signaling 13, 14, 15. Hence, we investigated whether treatment of cells at the ANE stage with BMPs would lead to increased expression of eye field features.

**Figure 2:**
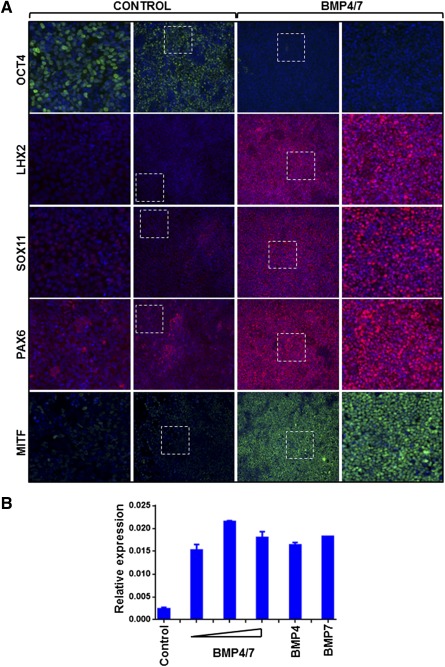
Stage 1: induction of eye field from human embryonic stem cells (hESC). **(A):** Two days’ postseeding, SHEF1 hESC were treated with Dulbecco's modified Eagle's medium KSR‐XF alone (Control) or Induction Medium 1 for 4 days, followed by Induction Medium 2 for 3 days (BMP4/7). Representative images showing immunocytochemistry for the pluripotency marker OCT4 and eye field markers LHX2, SOX11, PAX6, and MITF are shown. Dotted squares indicate the region that is magnified in the adjacent panels. Images are captured at ×10 magnification. **(B):** Quantitative polymerase chain reaction was carried out to measure expression of *Mitf* transcript in SHEF1 hESC treated with Induction Medium 1 alone (Control) or Induction Medium 1 supplemented with BMP4/7 (50 ng/ml, 100 ng/ml, 200 ng/ml; shown by triangle in order of ascending concentration); BMP4 (200 ng/ml); or BMP7 (200 ng/ml) (*n* = 3, ±SD). Abbreviation: BMP, bone morphogenetic protein.

To initiate differentiation, we seeded SHEF1 hESC as single dissociated cells. After 2 days (day 2), when the cells reach 80%–90% confluence, the culture medium was changed to Induction Medium supplemented with SB and LDN (Induction Medium 1). Dual SMAD inhibition was maintained for 4 days. At this point, that is, at day 6, LDN/SB were withdrawn, and the cells were treated with the BMP4/7 recombinant heterodimer (Induction Medium 2) for a period of 3 days. At day 9, immunocytochemistry was carried out to assess expression of key eye‐field transcription factors. There was a marked downregulation of the pluripotency marker OCT4 together with uniform induction of eye field markers such as LHX2, SOX11, PAX6, and MITF upon this sequential inhibition of Activin/BMP pathways followed by BMP activation, in comparison with noninduced cells (Fig. 2A). Similar results were obtained using a hiPSC line (SendaiF) as the starting material (supplemental online Fig. 1). To determine whether the observed effect was specific to the BMP4/7 heterodimer, we tested the individual BMP4 and BMP7 proteins in parallel with the BMP4/7 heterodimer and measured expression of *Mitf* transcript. Similar results were seen under all conditions (Fig. 2B) and 100 ng/ml BMP4/7 heterodimer was used for the remainder of the work, because it has been shown to be more potent for BMP signaling activation in other systems 20, 21.

### Efficient Differentiation of the Eye Field Toward RPE Requires Replating in the Presence of Activin A

**Figure 3:**
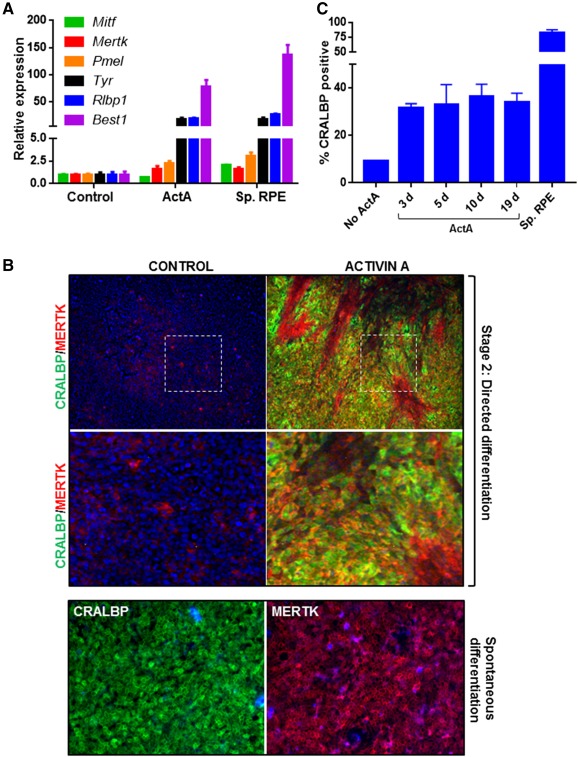
Stage 2: Generation of a mixed RPE population. **(A):** Two days’ postseeding, SHEF1 human embryonic stem cells were treated with Induction Medium 1 for 4 days, followed by Induction Medium 2 for 3 days. At day 9, cells were replated in Dulbecco's modified Eagle's medium (DMEM) KSR‐XF alone (Control) or Induction Medium 3 (ActA) for 19 days. Quantitative polymerase chain reaction was used to measure expression of a panel of RPE markers. RPE generated by using spontaneous differentiation (Sp. RPE) were used for comparison (*n* = 3, ±SD). **(B):** Representative images showing immunocytochemistry for CRALBP and MERTK in cells derived by directed differentiation and cultured as in panel A. Dotted squares indicate the region that is magnified in the panels below. RPE generated by spontaneous differentiation were used for comparison. Images are captured at ×10 magnification. **(C):** Quantification of CRALBP immunocytochemistry at day 28 in which cells during stage 2 were treated with DMEM KSR‐XF (Control) or Induction Medium 3 (ActA) for 3 days, 5 days, 10 days, or 19 days. For fewer than 19‐day ActA treatments, DMEM KSR‐XF was used for the remainder of the 19‐day period. RPE derived by using spontaneous differentiation (Sp. RPE) were used for comparison (*n* = 3, ±SD). Abbreviations: ActA, Activin A; d, days; RPE, retinal pigment epithelium; Sp. RPE, spontaneous differentiation retinal pigment epithelium.

Once the eye field develops, it is further specified toward the RPE lineage by Activin A (ActA) signaling, which is thought to be secreted by the mesenchyme neighboring the optic vesicle 16, 22. Indeed, some differentiation protocols have shown that treatment with ActA promotes RPE differentiation 9, 23. Therefore, to further direct the cells at prospective eye field stage toward the RPE lineage, we treated cells with ActA to mimic signaling cues from the extraocular mesenchyme. By day 9, the cells had become highly confluent; therefore the treatment with ActA was coupled with dissociation of the cells and replating on new matrix‐ coated surfaces. Cells were exposed to 100 ng/ml ActA (Induction Medium 3) for a period of 19 days (i.e., day 28 postseeding of pluripotent cells). At this point, cobblestone‐shaped cells resembling RPE could be seen in a mixed population (supplemental online Fig. 2A), and therefore, quantitative polymerase chain reaction (PCR) was carried out to measure transcript expression of RPE markers. ActA treatment following replating led to an induction of RPE genes such as *Mertk*, *Pmel*, *Tyr*, *Rlbp1*, and *Best1* (Fig. 3A). The expression of RPE genes was similar to levels in mature RPE, derived using an in vitro spontaneous differentiation method for RPE generation 18. These cells used for the purpose of comparison are herein referred to as “spontaneous RPE.” In order to verify that the transcript expression pattern was consistent with protein levels, we performed immunocytochemistry for MERTK and CRALBP. This demonstrated that although RPE induction could be detected, it was not uniform over the entire well (Fig. 3B). Therefore, RPE cells were present, albeit in a heterogeneous population. A similar result was seen using SendaiF hiPSC as the starting population (supplemental online Fig. 2B). We further demonstrated that the differentiation process was successful when using multiple different extracellular matrices for coating. Induction of cells with CRALBP expression was observed, regardless of whether cells were seeded on Matrigel or Fibronectin during stage 1 and replated onto Matrigel or CELLStart during stage 2 (supplemental online Fig. 2C). We also tested the extent of ActA exposure required for RPE induction. Cells replated at day 9 were treated with ActA for 3, 5, 10, or 19 days, and immunocytochemistry was carried out for CRALBP expression. There was no difference in percentage of CRALBP positive cells in cultures treated with differing lengths of ActA exposure, indicating that a 3‐day exposure was sufficient for RPE formation (Fig. 3C). This quantification also demonstrated that approximately 40%–50% of total cells were positive for CRALBP expression at this stage in comparison with >85% positive expression in spontaneous RPE, providing further support that RPE cells were present in a mixed population.

### Generation of Functional RPE Cultures Following a Second Replate Step

**Figure 4:**
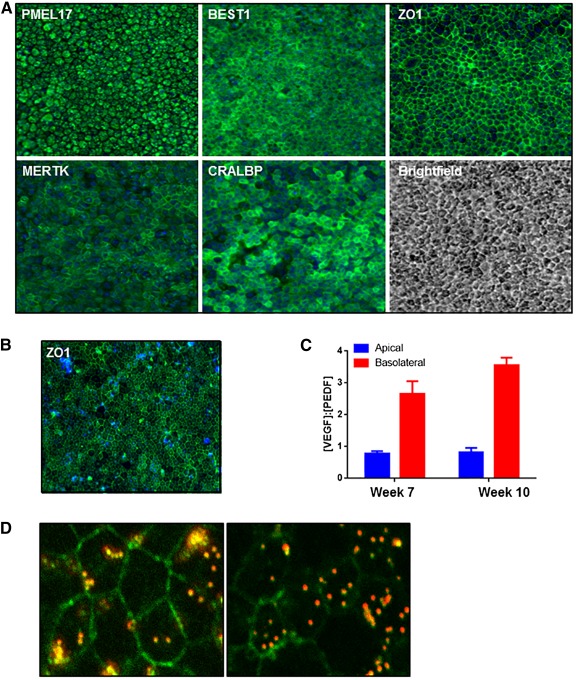
Stage 3: Generation of a homogenous and functional retinal pigment epithelium (RPE) population. **(A):** Two days’ postseeding, SHEF1 human embryonic stem cells were treated with Induction Medium 1 for 4 days, followed by Induction Medium 2 for 3 days. At day 9, cells were replated in Induction Medium 3 for a period of 19 days. At day 28, cells were replated in Dulbecco's modified Eagle's medium KSR‐XF and cultured for a period of 14 days. Representative images showing immunocytochemistry for indicated RPE markers are shown. Images are captured at ×10 magnification. **(B):** Cells cultured as in panel A were further dissociated and replated on transwells and cultured for a period of 10 weeks. A representative image showing en face immunocytochemistry for ZO‐1 is shown. Images are captured at ×20 magnification. **(C):** Spent medium from transwells described in panel B was collected from the top and bottom chambers and quantified for vascular endothelial growth factor (VEGF) and pigment epithelium‐derived factor (PEDF) concentration. The ratio of [VEGF]:[PEDF] was quantified in media from the two compartments (*n* = 3, ±SD). **(D):** Representative confocal images showing phagocytosis of fluorescent bead (red) by RPE. ZO‐1 immunocytochemistry (green) shows the cell edge, and the presence of bead within the cell boundary indicates internalization by phagocytosis. Images are captured at ×63 magnification. Abbreviations: PEDF, pigment epithelium‐derived factor; VEGF, vascular endothelial growth factor.

Previous reports have suggested that the process of dissociation and serial replating of mixed RPE cultures leads to purification of non‐RPE cells and the generation of pure RPE cultures 24. Therefore, we undertook the approach of passaging the heterogeneous RPE population generated at day 28 with a view toward increasing purity. Immunocytochemistry for mature RPE markers BEST1, PMEL17, MERTK, CRALBP, and ZO1 was carried out 14 days postreplating, that is, at day 42 of the protocol. Qualitative analysis of protein expression showed the presence of RPE cells with high purity after this second replating step (Fig. 4A). Similar results were obtained by using two different hESC (SHEF1 and WA26) and hiPSC (AD3.1 and SendaiF) cell lines as the starting population, with cobblestone‐shaped morphology (supplemental online Fig. 3A) and pigmentation typical of RPE cells visible after the second replate (supplemental online Fig. 3B). This shows that the directed differentiation method can be broadly applied across different pluripotent cells.

A key feature of RPE is their capability to polarize and secrete VEGF and PEDF in a vectorial manner 25, 26. This polarized secretion of PEDF apically and VEGF basolaterally is an important characteristic of healthy mature RPE cells in vivo. Apical secretion of PEDF acts to regulate the structural integrity of the retina by inhibiting invasive choroidal neovascularization, and basolateral secretion of VEGF, an angiogenic factor, supports the integrity of the choriocapillaris 2. Therefore, we tested whether RPE derived with our directed differentiation protocol secreted VEGF and PEDF in the expected fashion. Cells in stage 3 cultured for a period of 14 days were replated onto transwell inserts to enable polarization, and spent medium was sampled from the apical and basal compartments to quantify VEGF and PEDF levels. En face staining of the transwell insert for the tight junction protein ZO1 showed that the RPE monolayer was confluent and had a cobblestone‐like architecture (Fig. 4B). Measurement of the secreted factors at weeks 7 and 10 postseeding showed a (VEGF):(PEDF) ratio < 1 from the apical compartment and >1 in the basal compartment (Fig. 4C), indicating higher PEDF secretion apically and VEGF secretion basolaterally, in keeping with the profile expected in healthy RPE. Furthermore, the cells were capable of phagocytosing fluorescently labeled beads (Fig. 4D), which serves as a surrogate measure of ability to phagocytose rod outer segments 27. We also observed the formation of dome‐shaped structures in the cell monolayer (supplemental online Fig. 3C), which is indicative of formation of a cellular barrier and directional fluid transport by the cells 28 and is another feature of native RPE. Taken together, these data demonstrate that functional RPE populations can be obtained following the protocol described in this study.

### Microarray Profiling Reveals Stages of RPE Development

In order to understand the identity of the cells being produced during the different phases of the differentiation protocol, we used microarrays to profile the genome‐wide gene expression levels from cultures at multiple time points alongside RPE derived by spontaneous differentiation. Each time point was assayed in triplicate to understand variability within the protocol.

**Figure 5:**
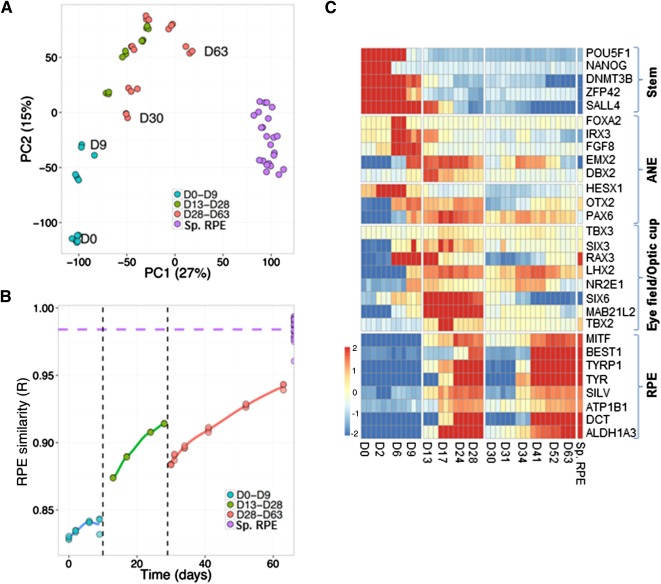
Transcriptome analysis of the directed differentiation protocol. **(A, B):** Human embryonic stem cells were cultured as described in Figure 4A, and samples were collected at different time points of the differentiation protocol. Similarity between samples at different points of the protocol is shown by principal components analysis **(A)** and Pearson's correlation coefficient **(B)**. **(C):** Microarray heatmap showing expression of markers representative of different stages of retinal pigment epithelium differentiation. The *x*‐axis refers to samples at different time points of directed differentiation. Abbreviations: ANE, anterior neuroectoderm; D, day; PC1, first principal component; PC2, second principal component; RPE, retinal pigment epithelium; Sp. RPE, RPE generated by spontaneous differentiation.

First, we performed PCA to reveal the relationship between samples on a global level (Fig. 5A). The close clustering of samples from the same time point demonstrates the high level of molecular reproducibility produced by the protocol. The first principal component (PC1) accounts for 27% of the gene expression variation in the data and appears to correspond largely to the difference between the differentiating cultures and the spontaneous RPE samples. Encouragingly, the differentiating samples became increasingly similar to the spontaneous RPE over time, as measured by their PC1 score. An alternative measure of global sample similarity is the Pearson's correlation coefficient (*R*) of the genome‐wide expression levels. Using this metric, we observed a steady increase in the global similarity over time within each phase, accompanied by a significant jump in similarity during stage 2 and a drop followed by recovery during stage 3 (Fig. 5B). A potential explanation of this profile is discussed in the following section.

In order to investigate the expression profiles more closely, we turned our attention to the expression of genes that may serve as markers of different cell populations within the in vitro cultures (Fig. 5C). As was expected, markers of the stem cell phenotype (e.g., *Pou5f1*, *Nanog*, *Dnmt3b*, *Zfp42*, and *Sall4*) were highly expressed during the early stage of differentiation, and their expression decreased with time. Treatment with LDN/SB committed cells toward the ANE and forebrain progenitor state, as is evidenced by expression of markers such as *Irx3*, *Hesx1*, *Fgf8*, *Otx2*, and *Pax6* 29, 30, 31. This was followed by expression of eye field transcription factors such as *Six3*, *Rax*, *Lhx2*, *Nr2e1*, and *Six6*, which have been shown to be essential for induction of the eye field in several models 32, 33. Expression of *Mab21l2* and *Tbx*, which are required for formation of the optic vesicle and optic cup structures 34, 35, further suggests commitment toward these fates. Finally, we observed expression of genes that are markers of the RPE fate (e.g., *Mitf*, *Best1*, *Tyr*, *Tyrp1*, *Pmel*, *Atp1b1*, *Dct*, and *Aldh1a3*) during the latter stages of stage 2 as well as during stage 3. The expression profile of representative genes was confirmed by quantitative PCR (supplemental online Fig. 4). By searching for other genes with expression patterns correlating with *Mitf*, we identified additional genes that serve as a good measure of the overall RPE molecular phenotype of cells within the culture (supplemental online Fig. 5A).

**Figure 6:**
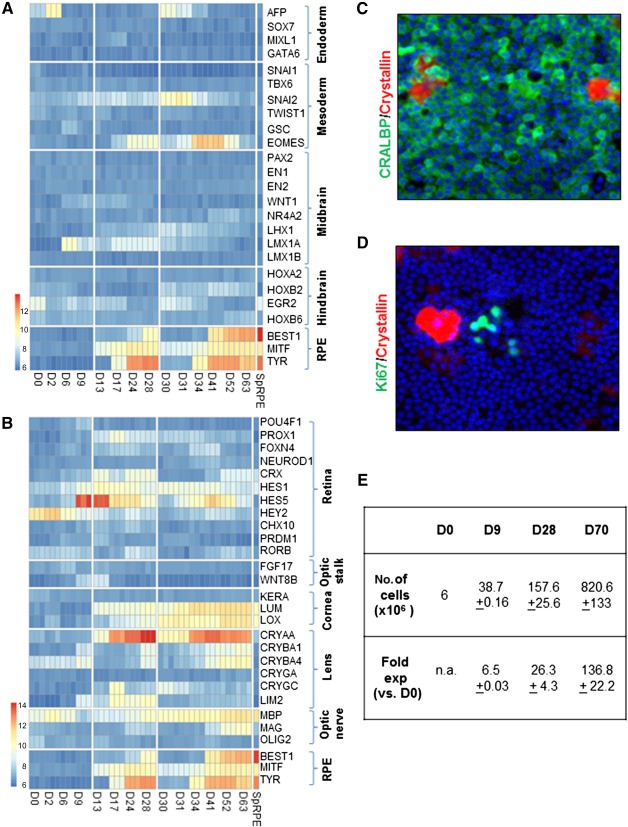
Specificity and efficiency of retinal pigment epithelium (RPE) differentiation. **(A, B):** Microarray heatmap showing expression of markers representative of different lineages and brain regions **(A)** and regions of the eye **(B)**. The *x*‐axis refers to samples at different time points of directed differentiation and the Sp. RPE samples refer to RPE generated by spontaneous differentiation. **(C, D):** Representative images for immunocytochemistry of CRALBP (green) and β‐crystallin (red) **(C)** and Ki67 (green) and β‐crystallin (red) **(D)** are shown. Images are captured at ×10 magnification. **(E):** Table summarizing yield and fold expansion of cells at various stages of the directed differentiation protocol. Abbreviations: D, day; exp, expansion; n.a., not applicable; RPE, retinal pigment epithelium; SpRPE, RPE generated by spontaneous differentiation.

In addition to induction of the RPE fate, we wanted to understand whether there were other non‐RPE lineages or cell types being formed as part of the differentiation process. Therefore, we looked at the expression of endoderm and mesoderm markers across the time course of RPE induction as well as markers characterizing the midbrain and hindbrain regions (Fig. 6A). In comparison with expression of RPE markers, we saw little evidence of formation of these other lineages, indicating that induction toward an eye‐specific RPE fate was taking place. We also examined the expression of markers of other cell types that originate from the eye field (e.g., the retina, optic stalk, cornea, lens, and optic nerve) (Fig. 6B). Expression of *Crya* transcripts encoding crystallins, which are markers of lens cells 36, could be observed at time points coinciding with expression of RPE genes. We further verified this by immunocytochemistry for β‐crystallin in RPE cells at day 48 of the directed differentiation protocol (Fig. 6C). Quantification of the positive cells showed that in comparison with >90% RPE measured by expression of CRALBP, <10% cells were positive for Crystallin, indicating that these constituted a minor proportion of the population. The Crystallin‐positive cells were nonproliferative, as is shown by the absence of Ki67‐positive staining (Fig. 6D). Taken together, these data indicate that directed differentiation toward the RPE lineage may lead to generation of a subpopulation of lens cells.

We were also intrigued by the observation that the initiation of stage 3 induced a decrease in RPE marker expression as well as in the overall molecular profile that made the culture temporarily less RPE‐like (Fig. 5B, 5C [RPE genes]). This suggests that the cells undergo a temporary phase, during which they lose their epithelial phenotype before regaining it during subsequent maturation. This resonates with our previous work demonstrating an epithelial‐mesenchymal‐epithelial transition in RPE derived by spontaneous differentiation 37. We have shown that when RPE are dissociated and plated, they undergo a period of proliferation during which they acquire mesenchymal characteristics and downregulate expression of epithelial genes. This is followed by a process of re‐epithelialization in which the cells reacquire their epithelial gene signature. Therefore, we hypothesized that a similar phenomenon might be occurring in the RPE cells purified and replated in stage 3 of directed differentiation. In order to address this hypothesis, we compared the gene expression profile during this stage with a dataset generated during dissociation and expansion of RPE derived from spontaneous differentiation, which captures the gene expression profiles during the epithelial‐mesenchymal‐epithelial transition 37. We found that the expression changes observed during the two processes are highly similar (*r* = .7; *p* < 1 × 10^−16^). Furthermore, a consistent downregulation of RPE markers such as *Mertk*, *Mitf*, *Rlbp1*, *Best1*, and *Tyr* and an upregulation of mesenchymal markers such as *Itga5*, *Msn*, *Cav1*, and *Serpine1* could be seen in cells during this stage of directed differentiation (supplemental online Fig. 5B). This suggests that when the RPE generated from stage 2 are replated, they undergo an epithelial‐mesenchymal transition‐like phenomenon before undergoing re‐epithelialization. This is consistent with the notion that the underlying biology and characteristics of the cells are similar, independent of whether they are generated by spontaneous differentiation or directed differentiation.

## Discussion

The use of pluripotent stem cell‐derived RPE holds great promise for cell replacement‐based therapies for AMD. Since the discovery of spontaneous RPE generation in hESC cultures more than 10 years ago 38, substantial effort has been invested in trying to develop more efficient RPE differentiation protocols that allow generation of cells at scale 10. This is because, although the spontaneous differentiation method successfully generates RPE, it is labor intensive and time consuming, requiring manual excision of pigmented foci, followed by their dissociation and expansion. Furthermore, limited understanding of how and what signaling cues lead to RPE generation in this approach makes it a variable and difficult‐to‐ control process that is not ideal for clinical application. Several protocols have since been developed to overcome the limitations of the spontaneous approach. Reports have described methods such as three‐dimensional (3D) cultures (e.g., neuroepithelial cysts as well as supplementation with growth factors and small molecules) 19, 23, 39, 40, 41, 42. However, to our knowledge, no study to date characterizes how the manipulations performed affect the molecular fate and dynamics of cells developing toward an RPE lineage using methods such as whole genome transcript analysis. Therefore, although RPE can be derived in vitro, clear understanding of the underlying stages of development is lacking.

Here, we describe a simple protocol, without the use of 3D culture or manual dissection‐based purification methods that leads to efficient generation of RPE with high yield and purity. We show that mature, functional RPE can be generated in a monolayer culture by stepwise modulation of Activin A and BMP signaling. Our method relies on the exogenous supplementation of only four additives: two small molecules (LDN‐193189 and SB‐431542) and two recombinant proteins (BMP4/7 and Activin A) in a temporal and sequential manner. The use of xeno‐free and defined culture medium such as TesR2 or E8 for the culture of pluripotent stem cells and DMEM KSR‐XF for differentiation—together with xeno‐free matrices such as Vitronectin for stem cells, human plasma Fibronectin for stage 1, and CellSTART for stages 2 and 3 of differentiation—eliminates the use of animal‐based or undefined products. Our method is transferable across different hESC and hiPSC lines and requires minimal cell line specific optimization. We have also tested its robustness across multiple operators and obtained identical results. In order to estimate the yield of cells using this protocol, we have quantified the number of cells obtained at the end of each phase. A starting culture of 6 × 10^6^ hESC yields more than 8 × 10^8^ RPE under the described seeding densities, which is equivalent to an expansion of approximately 136‐fold (Fig. 6E). This can potentially be further increased if seeding densities are altered and the RPE are expanded through additional passage. This high yield of cells can be put into better context by considering that a normal human adult eye contains 4–6 × 10^6^ RPE 43 and current cell replacement therapeutic approaches for treating AMD use a dose of 0.2 × 10^6^ RPE cells per eye 4. Therefore, the use of the described protocol has the potential to sustain manufacturing of the RPE cell product for treating a large number of patients even when executed at a comparatively modest scale, which is advantageous in terms of keeping the time, labor, and financial outlay as low as possible.

In addition to developing a robust protocol, we characterize the cells generated at every stage by performing whole genome transcript profiling across the time course of induction. This allows us to map the developmental fate of RPE in vitro as well as define the dynamics of the differentiation process. In doing so, we validate the role of SMAD inhibition, BMP, and Activin A pathways in RPE development, and it is extremely encouraging to note that cells follow a developmental path that would occur during normal ontogenesis in vivo. Such characterization has been lacking in all protocols described to date, including spontaneous differentiation relying on undefined signaling cues or directed differentiation using manipulation of signaling pathways. As a result, not only is there limited understanding of the differentiation process itself but also of the identity of non‐RPE “impurities” that might be present remains unknown. We show that pluripotent cells transition through anterior neuroectoderm, eye field, and optic vesicle/cup stages before giving rise to RPE cells and that there is little evidence to suggest formation of endoderm, mesoderm, midbrain, or hindbrain cell types. We detect a minor population of Crystallin‐positive cells, indicative of a lens phenotype, being generated together with RPE. This is not surprising, given that RPE and lens share common progenitors and RPE are known to transdifferentiate into lens in certain organisms 44, 45, 46. Our demonstration that these cells are nonproliferative allays concerns about the presence of undesirable proliferation in our RPE population, and further manipulation of signaling pathways required for lens development 47 may help to suppress the formation of this cell type. The transcriptomic analysis presented in this report enables knowledge of markers that are expressed at specific time points, which can be used to design in‐process controls for a manufacturing process or enable quality‐by‐design strategies for process development. Furthermore, the two replating steps at day 9 and day 28 offer potential opportunities to introduce cryopreservation steps that can offer a means to pause the differentiation process or allow a window to sample cells for quality control, both of which are desirable attributes for developing cell products. Finally, we note that the gene expression analysis indicates that the level of expression of RPE genes is higher in cells derived from spontaneous differentiation in comparison with directed differentiation. This may be due to the much longer time scales of spontaneous differentiation, which can be around 4–6 months postseeding pluripotent cells, followed by purification by manual excision and expansion. This may not be directly comparable to RPE derived by directed differentiation, which may be at an earlier stage of terminal differentiation and have not undergone additional purification to remove non‐RPE cells for, as an example, the Crystallin‐positive lens‐type cells as identified in our study. Further work comparing the function of RPE derived from spontaneous and directed differentiation in an in vivo setting is needed to clarify whether the difference in gene expression translates to a difference in quality or function.

In summary, we show that we can efficiently direct pluripotent stem cells toward retinal pigment epithelium fate by using a simple directed differentiation method. The high‐yield, short‐time scale, and xeno‐free culture conditions make it adaptable for use under GMP conditions for clinical applications. In addition, it can be applied to generate RPE for screening, modeling of retinal pathophysiologies, and further understanding of the AMD diseased state, potentially opening up more avenues for therapeutic intervention.

## Author Contributions

P.C.: conception and design, collection and/or assembly of data, data analysis and interpretation, manuscript writing, final approval of manuscript; H.B. and B.S.: collection and/or assembly of data, data analysis and interpretation, final approval of manuscript; A.G.: data analysis and interpretation, final approval of manuscript; I.L. and J.S.: collection and/or assembly of data, final approval of manuscript; J.K.: provision of study material, final approval of manuscript; P.J.W.: conception and design, final approval of manuscript.

## Disclosure of Potential Conflicts of Interest

P.C. was a compensated employee of Pfizer Ltd., is co‐inventor on a patent with Pfizer Ltd., and has stock options with Pfizer Ltd. H.B. has compensated employment, is co‐inventor on a patent, and has compensated research funding. A.G. is an employee of and has stock in Pfizer Ltd. B.S. was an employee of Pfizer Ltd. and is co‐inventor on a patent. I.L. and J.S. were compensated employees of Pfizer Ltd. J.K. was an employee of and has stock in Pfizer Ltd. P.J.W. was an employee of Pfizer Ltd. and is co‐inventor on a patent.

## Supporting information

Supporting Information
