# Supplementary material for: Directing Differentiation of Pluripotent Stem Cells Toward Retinal Pigment Epithelium Lineage
Source: Stem Cells Transl Med. 2016 Aug 24;6(2):490–501. doi: 10.5966/sctm.2016-0088 (PMC5442825; doi:10.5966/sctm.2016-0088)
Supplement: Supplementary file 1 — Supporting Information [file SCT3-6-490-s001.pdf]

Supplemental Figures – Choudhary et al.

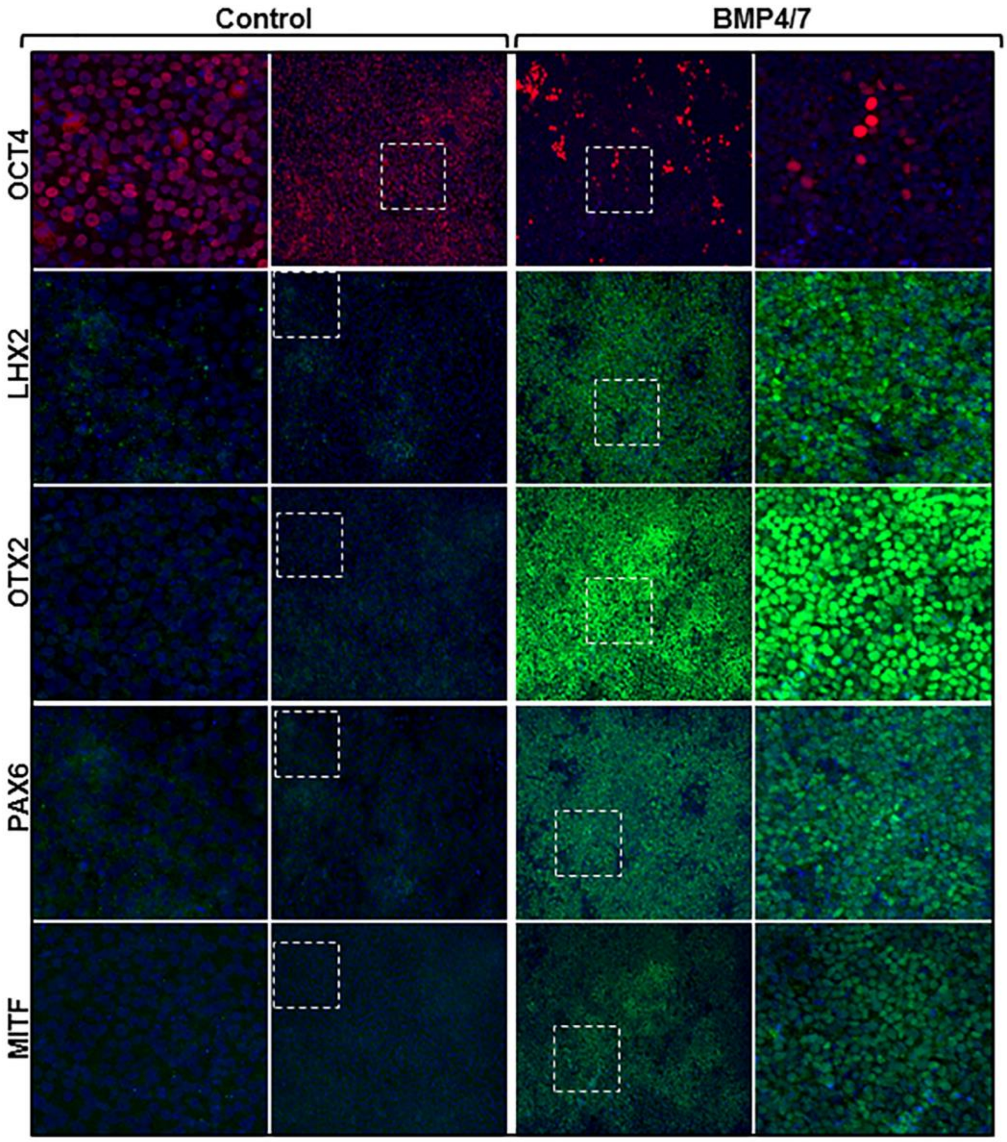

Figure S1: Stage 1- differentiation of hiPSC

Two days post seeding, SendaiF hiPSC were treated with DMEM KSR-XF alone (Control) or Induction Medium 1 for 4 days followed by Induction Medium 2 for 3 days (BMP4/7). Representative images showing immunocytochemistry for the pluripotency marker OCT4 and eye field markers LHX2, OTX2, PAX6 and MITF are shown. Dotted squares indicate the region that is magnified in the adjacent panels. Images are captured at 10x magnification.

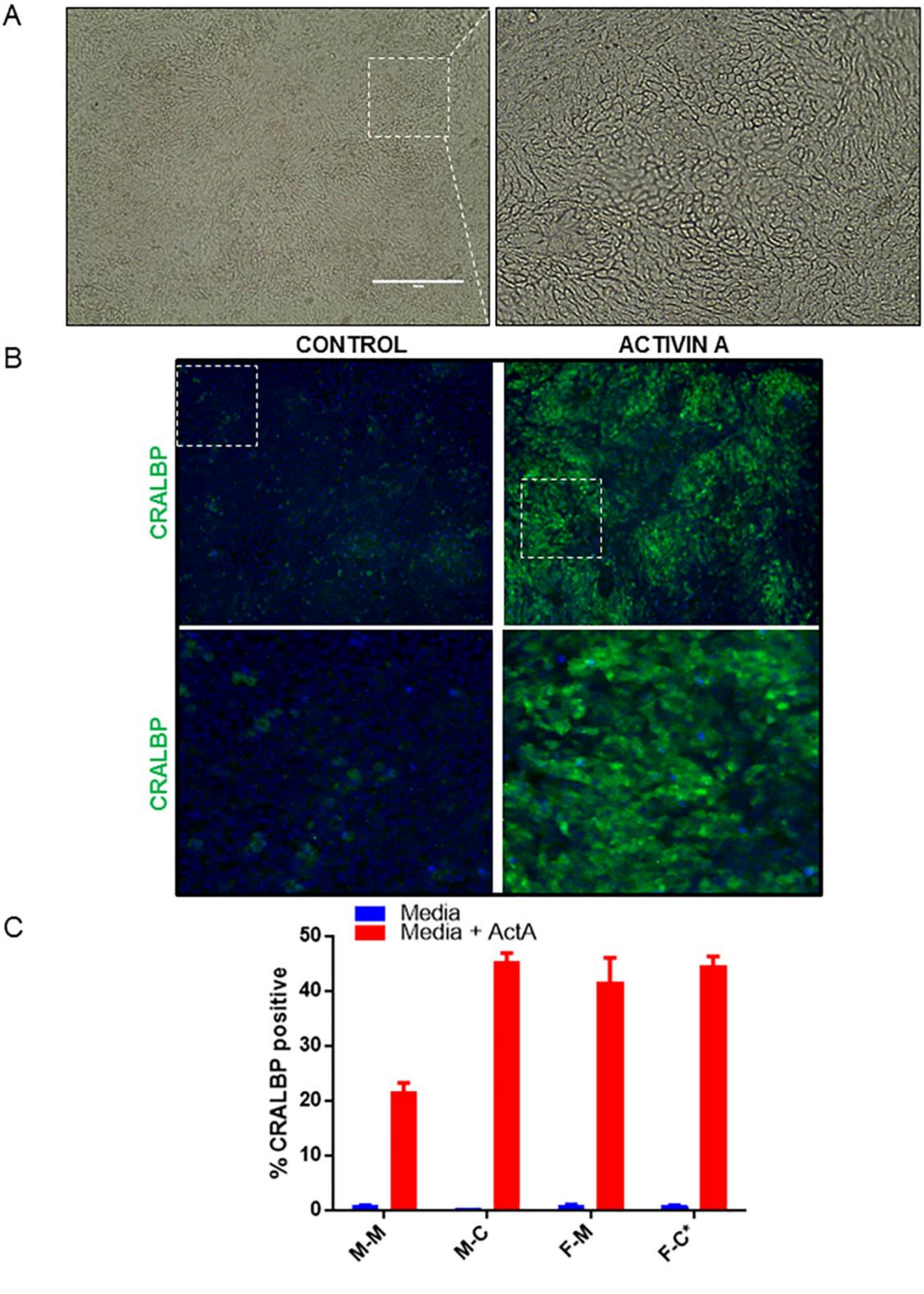
A
B
CONTROL
ACTIVIN A
CRALBP
CRALBP
C
Media
Media + ActA
% CRALBP positive
50
40
30
20
10
0
M-M
M-C
F-M
F-C*

Figure S2

A. Representative brightfield image showing presence of cobblestone shaped cells in a mixed population at Day 28. Dotted squares indicate the region that is magnified in the adjacent panel. Images are captured at 20x magnification. Scale bar indicates 200µm.

B. Two days post seeding, SendaiF hiPSC were treated with Induction Medium 1 for 4 days followed by Induction Medium 2 for 3 days. At Day 9, cells were replated in DMEM KSR-XF alone (Control) or Induction Medium 3 (Activin A) for 19 days. Representative images showing immunocytochemistry for CRALBP are shown. Dotted squares indicate the region that is magnified in the panels below. Images are captured at 10x magnification.

C. Test of matrices. Quantification of immunocytochemistry for CRALBP in cells where Stage 1 is carried out on Matrigel (M) or Human Plasma Fibronectin (F) and Stage 2 on CELLStart (C). * denotes the xeno-free combination of Fibronectin in Stage 1 followed by CELLStart in Stage 2. (n=3, ± S.D)

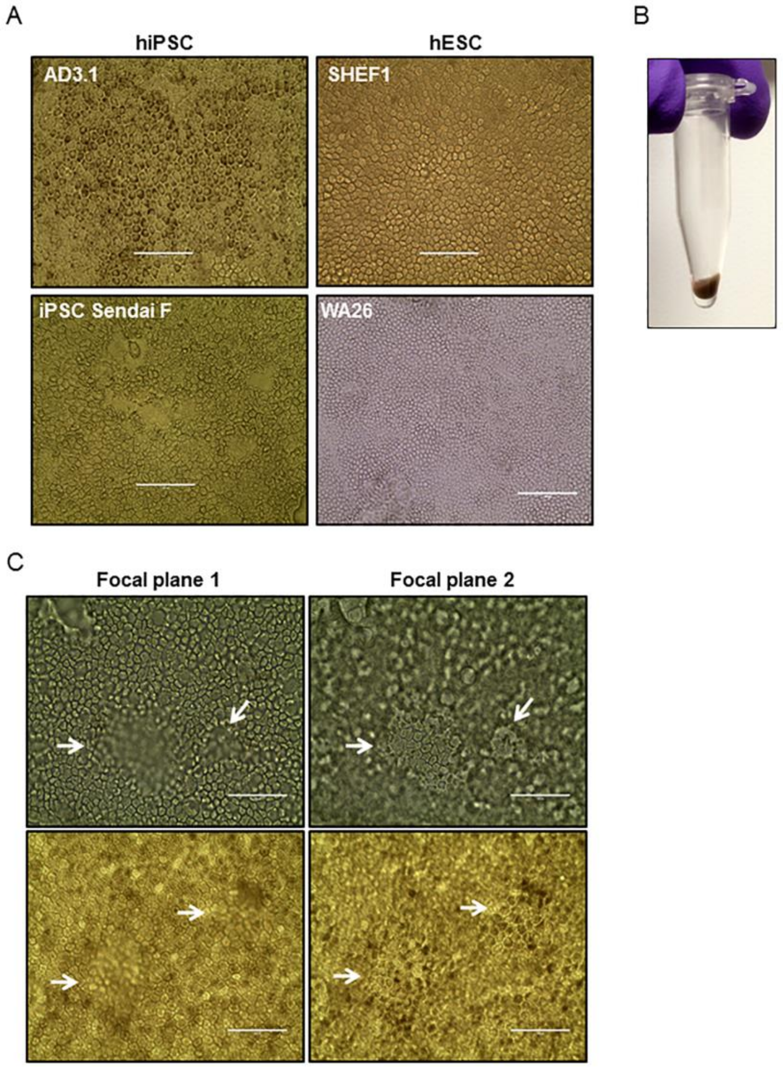

Figure S3

A. Representative brightfield images showing cobblestone architecture in cells generated using the directed differentiation protocol after Stage 3. The starting material used is either hiPSCs (AD3.1, SendaiF) or hESCs (SHEF1, WA26). Images are captured at 40x (AD3.1, SendaiF, SHEF1) or 20x (WA26) magnification. Scale bar indicates 100µm (AD3.1, SendaiF, SHEF1) or 200µm (WA26)

B. Representative image showing pigmentation in a pellet of cells generated by the directed differentiation protocol

C. Representative brightfield images showing the formation of a fluid-filled domes by the SHEF1 hESC-RPE (top) and AD3.1 hiPSC-RPE (bottom). The domes are marked by white arrows and the images are captured in in two different focal planes. Images are captured at 40x magnification. Scale bar indicates 100µm.

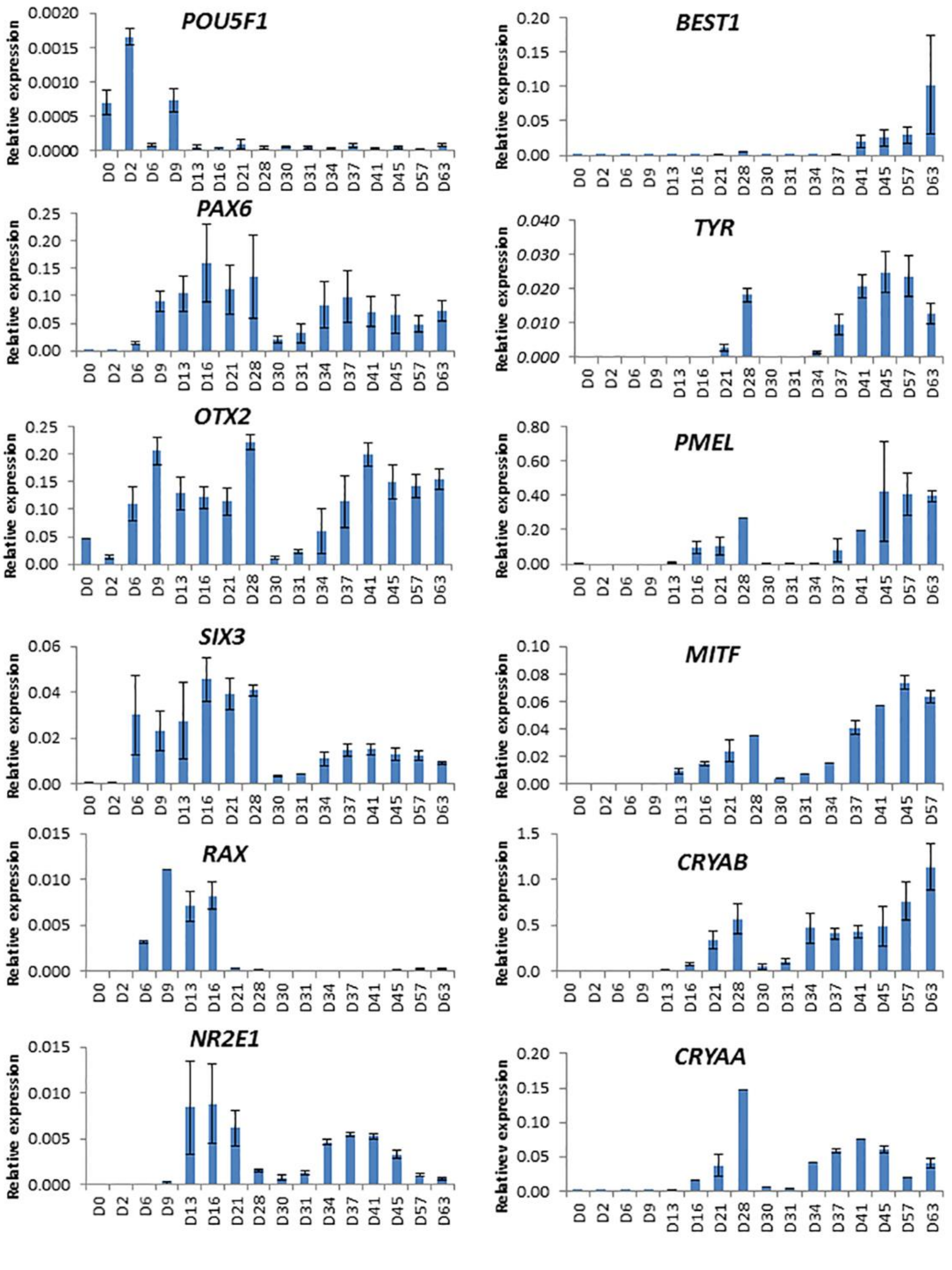

Figure S4:

qPCR based measurement of transcript expression of a panel of markers in samples collected at various timepoints of differentiation. *GAPDH* and *ACTB* are used as housekeeping genes. Bars represent Mean + SD (n=3-6). P<0.05 (Student's t-test).

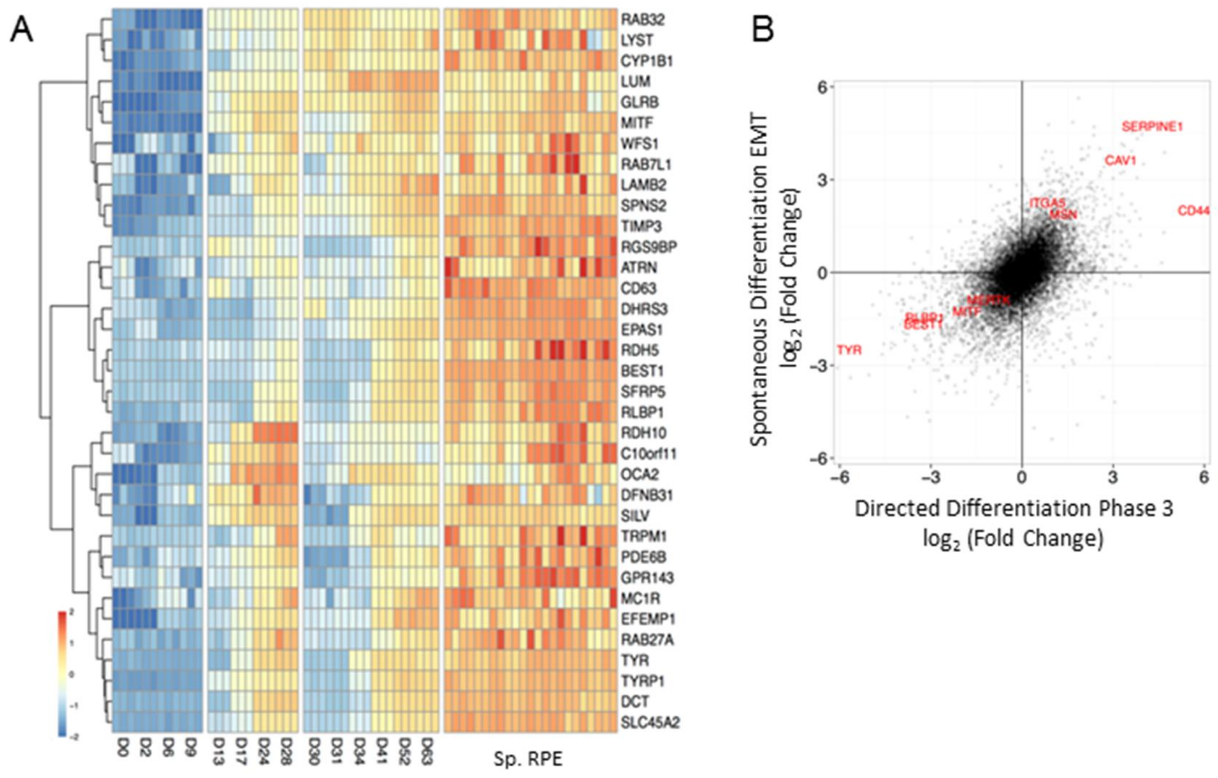

Figure S5:

A. Microarray heatmap showing expression of genes with a profile similar to RPE.

B. Scatterplot showing fold changes for indicated genes observed during Stage 3 of directed differentiation and EMT in RPE obtained from spontaneous differentiation.
